# Supplementation of Maize- and Cowpea Seed-Based Artificial Diets with Diverse Pollen Sources Affects the Demographic Features of *Leucania loreyi* (Duponchel, 1827) (Lepidoptera: Noctuidae)

**DOI:** 10.3390/insects17030307

**Published:** 2026-03-12

**Authors:** Maryam Jafari, Seyed Ali Hemmati, Lukasz L. Stelinski

**Affiliations:** 1 Department of Plant Protection, Faculty of Agriculture, Shahid Chamran University of Ahvaz, Ahvaz 61357-43311, Iran; maryamjafari3735@gmail.com; 2 Department of Entomology and Nematology, Citrus Research and Education Center, University of Florida, Lake Alfred, FL 33850, USA; stelinski@ufl.edu

**Keywords:** maize caterpillar, pollen supplementation, life table parameters, mass-rearing, demographic traits, population growth

## Abstract

*Leucania loreyi* (Duponchel, 1827) is a major lepidopteran pest that infests a wide range of crops worldwide. Effective mass production of insects for pest management programs depends on the availability of suitable artificial diets. Here, we evaluated 14 artificial diets (D1–D14) formulated from maize or cowpea seeds (19.5 g) plus standard diet components and supplemented with 1 g of pollen from different sources (rapeseed, date palm, maize, common hollyhock, saffron, and honey bee), along with control diets. We assessed their effects on demographic traits of *L. loreyi*. The maize seed–maize pollen diet (D3) and the cowpea seed–maize pollen diet (D10) produced the shortest developmental times (37.53 and 38.10 days, respectively), whereas the maize seed–saffron pollen (D5) and cowpea seed–saffron pollen (D12) diets resulted in the longest development (45.83 and 45.56 days, respectively). Diet also D3 yielded the shortest adult and total pre-oviposition periods (APOP and TROP), the greatest female longevity, and the highest fecundity and net reproductive rate (*R*_0_) (801.69 and 88.69 offspring, respectively). In contrast, diet D12 produced the lowest fecundity and *R*_0_ (339.73 and 68.15 offspring, respectively). Consistent with these patterns, D3 generated the highest intrinsic rate of increase (*r*) and finite rate of increase (*λ*), while diets D5 and D12 were associated with lower population growth rates. Cluster analysis further identified D3 as the most nutritionally favorable formulation under our experimental conditions, supporting its potential utility for large-scale *L. loreyi* rearing.

## Simple Summary

The maize caterpillar, *Leucania loreyi* (Duponchel, 1827) (Lepidoptera: Noctuidae) has emerged as a major global agricultural pest. Optimization of its rearing would facilitate its application in management strategies. In this study, we supplemented maize- or cowpea seed-based artificial diets with pollen from six different sources—rapeseed, date palm, maize, common hollyhock, saffron, and honey bee—to examine how these dietary combinations affect insect development and population growth. Supplementation of cowpea seed-based diets—and, more notably, maize seed-based diets—with maize pollen yielded the most favorable demographic outcomes for *L. loreyi*, including shortened developmental durations and enhanced reproductive and life table parameters, confirming maize pollen as the most suitable dietary supplement for rearing this insect. Inclusion of saffron pollen in maize- and cowpea-seed-based diets proved to be a less suitable supplement for promoting *L. loreyi* population growth, whereas the other pollen types produced moderate effects. Overall, the results indicate that an artificial diet based on maize seed and supplemented with maize pollen is the most suitable for rearing *L. loreyi* larvae and can be effectively used for large-scale insect production.

## 1. Introduction

The maize caterpillar, *Leucania* (=*Mythimna*) *loreyi* (Duponchel, 1827) (Lepidoptera: Noctuidae) is a widely occurring noctuid species that causes recurrent outbreaks in tropical and semi-tropical regions of Asia, Australia, and Africa [1,2]. As a polyphagous species, it attacks a wide array of agricultural crops [3,4] and can inflict serious economic damage when population densities exceed established thresholds [2]. Adults exhibit a strong capacity for long-distance dispersal [5,6,7], while the nocturnal larvae often migrate collectively between fields in search of food [6]. Early instars preferentially feed on young leaves, whereas later instars expand feeding to additional plant tissues, including stems and grains [2]. The pest’s development, survival, and population dynamics are strongly influenced by environmental conditions, with peak population performance reported during warm months (June-September), when temperatures are generally higher [8].

Research interest in *L. loreyi* has increased following recent outbreaks across multiple provinces in Iran, where favorable climatic conditions and the widespread cultivation of suitable host crops facilitate its survival and reproduction [4,8,9]. In the context of eco-friendly pest management strategies, Jafari et al. [4] evaluated the resistance of ten maize hybrids to *L. loreyi* and identified SC260 and SC706 as promising cultivars capable of suppressing pest populations in infested areas. Nevertheless, the development and evaluation of effective management approaches require the ability to produce and maintain large, stable laboratory populations of *L. loreyi* to support experimental research [4,10].

A critical requirement for laboratory insect rearing is the development of artificial diets that sustain the complete life cycle of target species under controlled conditions [10]. In recent years, low-cost, readily available plant-derived ingredients—particularly seeds and pollens—have attracted increasing attention because they can function both as sole food sources and as nutritionally rich components supplying amino acids, carbohydrates, lipids, vitamins, and minerals in formulated diets [11,12,13,14,15,16]. In addition, these materials provide feeding stimulants and host-plant-like chemical cues that enhance insect fitness traits such as survival, growth, fecundity, and longevity—attributes essential for efficient mass rearing [16,17,18,19].

Accordingly, numerous artificial diets have been developed for large-scale rearing of *L. loreyi* and other noctuid pests, supporting applications ranging from biological control and sterile insect technique programs to host plant resistance screening, nucleopolyhedrovirus production, push–pull strategies, and insecticide resistance studies [18,20,21,22]. Meridic diets containing pinto bean and soybean meal, for example, have been shown to enhance development and reproduction in *Spodoptera frugiperda* J.E. Smith (Lepidoptera: Noctuidae) [23] and were later validated as suitable for *L. loreyi* rearing [10]. More recently, seed-based and pollen-supplemented diets have been shown to significantly influence demographic parameters in noctuid species such as *Helicoverpa armigera* (Hübner) (Lepidoptera: Noctuidae) [15,18,19].

Despite the widespread use of seed-based ingredients in noctuid artificial diets, the combined inclusion of seeds and pollen within a single larval diet has received limited attention, particularly for *L. loreyi* [16,19,24]. Although pollen has been evaluated as a food source for adult *L. loreyi* females [1], its role as a dietary supplement during larval development remains poorly understood. Therefore, the objective of this study was to develop and evaluate seed-based artificial diets supplemented with different pollen sources for *L. loreyi* larvae. Specifically, maize seeds (the preferred host plant) and cowpea seeds were used as primary nutritional components, with pollen incorporated as a dietary supplement. Diet suitability was assessed by quantifying key demographic and life table parameters to generate baseline data for efficient laboratory rearing and to support development of a cost-effective artificial diet for large-scale production and pest management programs under controlled conditions.

## 2. Materials and Methods

### 2.1. Origin, Identification, and Rearing of Leucania loreyi

A laboratory colony of *L. loreyi* was established from larvae originally collected from infested maize fields in Behbahan County, Khuzestan Province, Iran (30°35′39″ N, 50°14′36″ E) in August 2024. Collected larvae were randomly assigned to 14 groups and reared on 14 different artificial diets under controlled-environment conditions [25 ± 1 °C, 60 ± 5% relative humidity, and a 16:8 h (L:D) photoperiod]. Emerged adults were maintained on a 10% honey solution. Species identification was confirmed through morphological examination of adult genitalia following the criteria described by Ben Jemâa et al. [2].

### 2.2. Seed and Pollen Sources

Seeds of maize (*Zea mays* L.) (Poaceae) (cultivar SC704) and cowpea (*Vigna unguiculata* L. Walp.) (Fabaceae) (cultivar Mashhad) were obtained from the Seed and Plant Improvement Research Institute, Karaj, Iran. Maize and cowpea were selected as base seed components because they are commonly used plant-derived ingredients in artificial diets for noctuid larvae and provide distinct nutritional profiles. Seeds were ground individually, sieved to obtain a fine powder, and used as primary ingredients in the preparation of artificial diets.

Pollen from six sources—rapeseed (*Brassica napus* L.) (Brassicaceae), date palm (*Phoenix dactylifera* L.) (Arecaceae), maize (*Zea mays* L.), common hollyhock (*Althaea rosea* L.) (Malvaceae), saffron (*Crocus sativus* L.) (Iridaceae), and honey bee (*Apis mellifera* L. (Hymenoptera, Apidae); a naturally mixed pollen collected from multiple plant species)—was used in this study. These pollens were selected to represent plant species commonly encountered by *L. loreyi* in agroecosystems and to provide variation in the composition of primary and secondary metabolites. Pollens of rapeseed, date palm, maize, and common hollyhock were collected from plants grown in research fields on the campus of Shahid Chamran University of Ahvaz, Ahvaz, Iran. Saffron and honey bee pollens were obtained from local producers within the same geographic region and during the same growing season. While honey bee pollen composition may vary temporally and spatially, using locally sourced honey bee pollen was intended to reduce variability and better represent natural feeding conditions. For pollen collection, reproductive structures of the respective plants were individually bagged and transported to the laboratory, air-dried, and gently shaken to release pollen grains into clean plastic containers. The collected material was then sieved to remove plant debris. All pollens were stored at 4 °C for short-term use or at −20 °C for long-term storage prior to incorporation into the artificial diets.

### 2.3. Diet Preparation

A total of 14 seed-pollen-based artificial diets (D1 to D14) were prepared for this study. The control diets consisted of standard and modified meridic formulations based on the Shorey and Hale [25] diet, with cowpea seed powder (diet D14) and maize seed powder (diet D7) serving as the primary components, respectively. The remaining 12 diets were derived from diets D7 and D14 by replacing 1 g of the corresponding seed powder with pollen from rapeseed, date palm, maize, common hollyhock, saffron, or honey bee. The pollen inclusion level (1 g; ~3.4% *w*/*w* of total dry ingredients) was set based on preliminary optimization trials to ensure diet homogeneity and larval acceptance and was chosen for standardization rather than to mimic natural feeding proportions. All other ingredients and their proportions in the base formulation were kept constant across diets. The complete composition of all experimental diets, including both major ingredients (seed powder and pollen) and minor ingredients (yeast, wheat germ, preservatives, agar, oil, and water), with quantities, is provided in Table 1. All synthetic chemicals used in this study were purchased from Sigma Chemical Co. (St. Louis, MO, USA).

### 2.4. Development of Leucania loreyi on Experimental Diets

For each diet, three independent cohorts (replicates) of 50 age-synchronized eggs (<24 h old) were used for life table analysis. They were obtained from adults that had been reared as larvae on the respective experimental diets. Eggs were gently transferred using a fine brush into plastic containers (15 cm diameter × 13 cm height), each containing a moistened cotton ball to maintain adequate humidity. Containers were maintained in a controlled-environment growth chamber set to 25 ± 1 °C, 60 ± 5% relative humidity, and a 16:8 h (L:D) photoperiod; conditions were automatically regulated and continuously monitored throughout the experiment. To minimize variation among containers, identical rearing containers were used, diets were prepared in a single batch for each treatment, and containers were randomly arranged and periodically repositioned within the chamber. Eggs were inspected daily to record hatching time and egg survival for each diet.

Upon hatching, neonate larvae were individually transferred to plastic Petri dishes (8 cm diameter × 1.5 cm height) with ventilation holes (1 cm diameter) covered with fine mesh. Each larva was considered a single biological replicate. Each individual was provided daily with approximately 1 cm^3^ of the assigned diet, which was replaced as needed, and reared separately until the prepupal stage. Larval survival and the durations of the larval and prepupal stages were recorded through daily observations. After pupation, each pupa was monitored daily until adult emergence to determine pupal developmental duration on each diet. Pupal sex was determined prior to the reproduction experiments based on the position and morphology of the genital opening, following Jiang et al. [26].

### 2.5. Reproduction of Leucania loreyi on Experimental Diets

Newly emerged male and female adults of *L. loreyi*, originating from the 50 individuals monitored during immature development, were randomly paired into multiple independent mating pairs per diet for assessment. of reproduction. Each pair was placed in an individual net-covered plastic container (25 × 30 cm) equipped with folded paper to serve as an oviposition substrate. Adults were provided with a 10% honey solution supplied on a cotton ball placed in a small plastic container (2.5 cm diameter × 3.5 cm height).

Egg production per female (fecundity) was recorded daily. The adult pre-oviposition period (APOP; time from adult emergence to first oviposition), total pre-oviposition period (TPOP; time from egg hatch to first oviposition), oviposition period, adult longevity (time from adult emergence to death), and total lifespan (time from egg hatch to death) were calculated for each dietary treatment. Adults were observed daily until all individuals had died.

### 2.6. Life Table of Leucania loreyi on the Studied Diets

The life-table experiment was conducted using a completely randomized design with 14 dietary treatments and 50 replicates per treatment. Life table parameters were estimated from individual-based data using the age-stage, two-sex life table methodology. Age-specific survival rates (*l_x_*) and age-specific fecundity (*m_x_*) were calculated from daily records of survival and egg production for all individuals within each cohort. These parameters were then used to estimate key life table metrics for *L. loreyi* reared on the experimental diets, including the intrinsic rate of increase (*r*), net reproductive rate (*R*_0_), gross reproductive rate (*GRR*), finite rate of increase (*λ*), and mean generation time (*T*), following Huang and Chi [27]. The age-specific survival (*l_x_*) and fecundity (*m_x_*) were calculated as follows:

(1) $$m_{x}=\frac{\sum_{j=1}^{k}S_{xj}f_{xj}}{\sum_{j=1}^{k}S_{xj}}$$

(2) $$l_{x}=\sum_{j=1}^{k}S_{xj}$$

where *k* is the number of stages, *S_xj_* (age-stage specific survival rate) is the age–stage–specific survival rate, defined as the probability that a newly oviposited egg survives to age *x* and stage *j*, and *f_xj_* (age-stage specific fecundity) is the age–stage–specific fecundity, expressed as the number of eggs produced by a female at age *x* and stage *j*. The intrinsic rate of increase (*r*) was estimated using an iterative bisection procedure to solve the Euler–Lotka equation.

(3) $$\sum_{x=0}^{\infty }\;e^{-r(x+1)}l_{x}m_{x}=1$$

The *R*_0_ was estimated as:

(4) $$R_{0}=\sum_{x=0}^{\infty }{l_{x}m}_{x}$$

with age indexed from zero in both formulae. The parameters of *GRR*, *λ*, and *T* were then estimated as: $GRR=\sum_{x=0}^{\infty }m_{x}$, $\lambda =e^{r}$, and $T=\frac{lnR_{0}}{r}$. The life expectancy of an individual at age *x* and stage *j* (*e_xj_*) was estimated as follows:

(5) $$e_{xj}=\sum_{i=x}^{\infty }\sum_{y=j}^{k}{S^{\prime }}_{iy}$$

where *S^′^_iy_* represents the probability that an individual at age *x* and stage *j* survives to age *i* and stage *y* [28,29].

### 2.7. Data Analysis

Demographic data were analyzed using the age–stage, two-sex life table approach [28,30] implemented in the TWOSEX-MSChart program [31]. Standard errors and variances for developmental, reproductive, and survival traits, as well as for life table parameters—including the intrinsic rate of increase (*r*), finite rate of increase (*λ*), gross reproductive rate (*GRR*), net reproductive rate (*R*_0_), and mean generation time (*T*)—were estimated using a bootstrap procedure with 100,000 resamples to ensure high precision. Differences among artificial diet treatments were evaluated using paired bootstrap tests at a 5% significance level, as commonly applied in demographic analyses based on age-stage, two-sex life table theory. Associations among key demographic parameters of *L. loreyi* across the experimental diets were assessed using Pearson’s correlation analysis to describe and visualize relationships in a heat map rather than to formally test statistical hypotheses. Relationships among dietary treatments were further explored using hierarchical cluster analysis (Ward’s minimum-variance method [32]) based on the demographic performance of *L. loreyi* on each diet. Clustering was used as an exploratory tool to visualize overall patterns, and no formal validation was performed.

## 3. Results

The development and survival of *L. loreyi* on the experimental artificial diets are summarized in Table 2. Developmental parameters differed significantly among the tested diets (*p* < 0.05). The shortest incubation period was observed on diet D3 (maize seed–maize pollen diet), whereas the longest incubation period occurred on diet D12 (cowpea seed–saffron pollen diet). Larval duration and total developmental time were shortest on diets D3 and D10 (cowpea seed–maize pollen diet) and were significantly prolonged on diets D5 (maize seed–saffron pollen diet) and D12. In contrast, the prepupal period was not significantly affected by diet. The pupal period, however, was significantly shorter on diets D3 and D10 and longest on diet D12. Pre-adult survival was highest on diet D3 (0.60), which was significantly greater than that observed on diet D11 (cowpea seed–common hollyhock pollen diet; 0.30) (*p* < 0.05).

Adult pre-oviposition period (APOP), total pre-oviposition period (TPOP), oviposition period, fecundity, longevity, and total lifespan of *L. loreyi* were all significantly influenced by the experimental diets (*p* < 0.05), with means ± SE presented in Table 3. The shortest APOP was observed on diet D3, whereas longer APOP values occurred on diets D1 (maize seed–rapeseed pollen-based diet) and D12. The lowest TPOP values were recorded on diets D3 and D10, while the highest TPOP occurred on diet D5. Oviposition duration varied among diets, ranging from 9.09 days on diet D5 to 11.89 days on diet D3. Females reared on diet D3 exhibited the highest fecundity and greatest longevity, whereas those reared on diet D12 showed the lowest values for both traits. Male longevity also differed significantly among diets, with the longest lifespan observed on diet D10 and the shortest on diet D5. Total lifespan was shortest for females on diet D10 and for males on diet D3.

**Figure 1:**
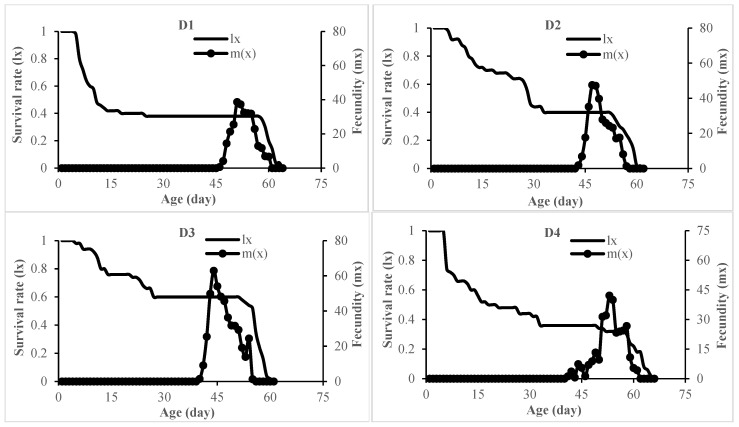

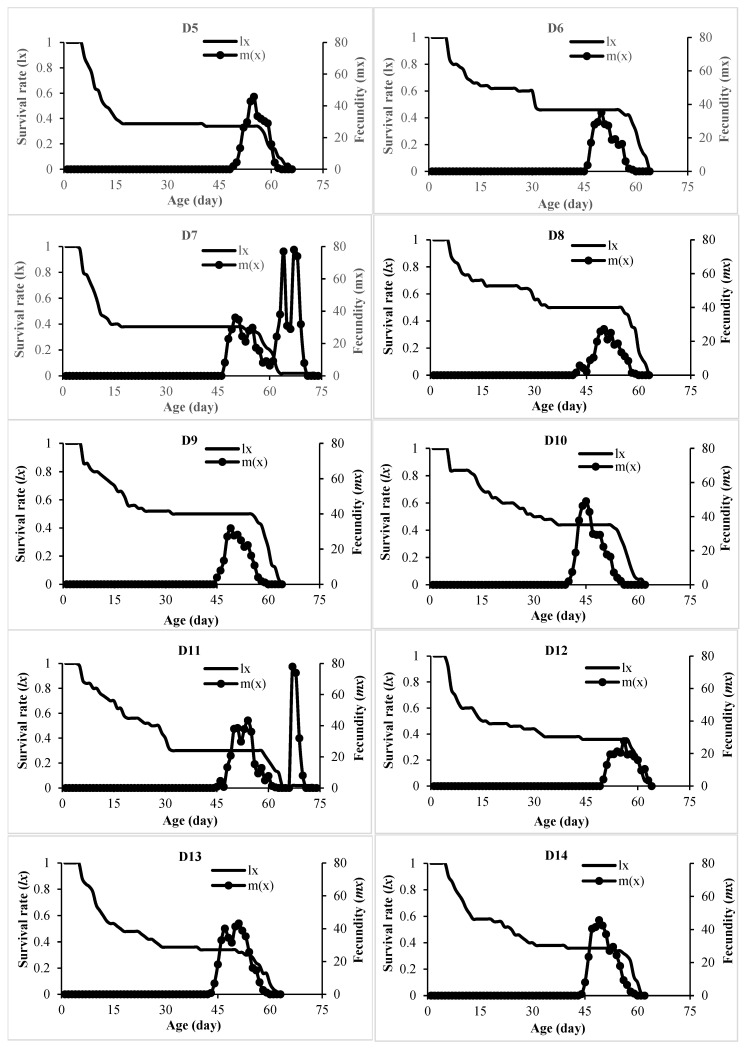
Age-specific survival rate (*l_x_*) and age-specific fecundity (*m_x_*) of *Leucania loreyi* reared on maize- and cowpea seed-based artificial diets supplemented with pollen from various sources. Diets D1–D6 consisted of maize seed combined with pollen from rapeseed, date palm, maize, common hollyhock, saffron, and honey bees, respectively. Diet D8–D13 consisted of cowpea seed combined with the same sequence of pollen sources. Diets D7 and D14 contained maize and cowpea seeds alone, respectively.

**Figure 2:**
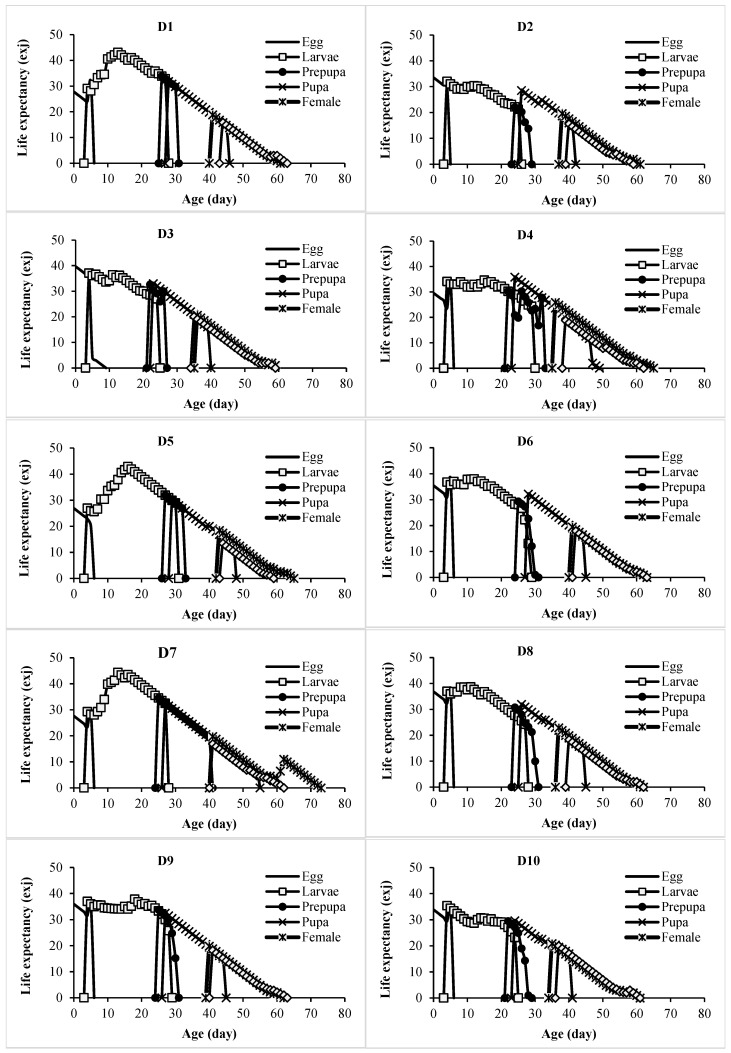

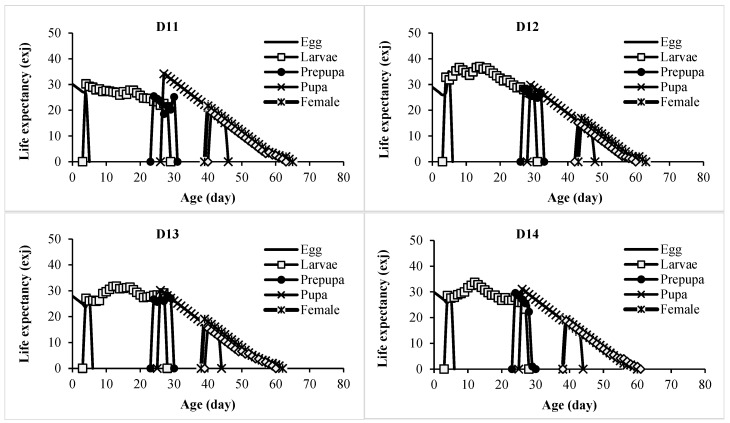
Age-stage specific life expectancy (*e_xj_*) of *Leucania loreyi* reared on maize and cowpea seed-based artificial diets supplemented with pollen from various sources. Diets D1–D6 consisted of maize seed combined with pollen from rapeseed, date palm, maize, common hollyhock, saffron, and honey bees, respectively. Diet D8–D13 consisted of cowpea seed combined with the same sequence of pollen sources. Diets D7 and D14 contained maize and cowpea seeds alone, respectively.

The age-specific survival rate (*l_x_*) and fecundity (*m_x_*) of *L. loreyi* across the experimental diets are shown in Figure 1 and indicate clear diet-dependent effects. Survival (*l_x_*) declined steadily with age, with cohort survival reaching zero between day 60 and day 73 across diets. Females initiated oviposition between day 39 and day 49. Peak age-specific fecundity (*m_x_*) ranged from 27.28 to 54.07 eggs per female per day and occurred between day 44 and day 55, after which daily egg production gradually declined and ceased between day 54 and day 69. Life expectancy (*e_xj_*) of a newly laid egg (Figure 2) also varied among diets, ranging from 26.92 to 39.70 days.

Life table parameters of *L. loreyi* differed significantly among the experimental artificial diets (*p* < 0.05), with detailed values presented in Table 4. The net reproductive rate (*R*_0_) was lowest on diet D12 (68.15 eggs per female) and substantially higher on diet D3 (288.36 eggs per female). The intrinsic rate of increase (*r*) and finite rate of increase (*λ*) reached their highest values on diet D3 but were markedly reduced in insects reared on diets D5 and D12. The gross reproductive rate (*GRR*) was more than twofold higher in individuals reared on diets D1, D3, and D7 (maize seed-based diets) compared with those reared on diet D12. Mean generation time (*T*) was shortest for individuals reared on diet D3, whereas longer generation times were observed on diets D5 and D12.

**Figure 3:**
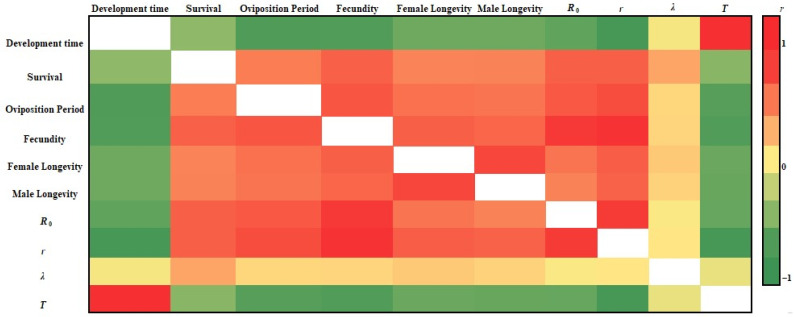
Heat map showing the relationships among major demographic parameters of *Leucania loreyi* reared on maize and cowpea seed-based artificial diets supplemented with diverse pollen sources. Color intensity reflects the magnitude and direction of correlations, with darker red indicating stronger positive correlations and darker green indicating stronger negative correlations. Abbreviations: *R*_0_, net reproductive rate; *r*, intrinsic rate of increase; *λ*, finite rate of increase; *T*, mean generation time.

The major demographic parameters were significantly intercorrelated, displaying both positive and negative associations (Figure 3). Development time was negatively correlated with survival (*r* = −0.571; *df* = 12; *p* = 0.033), oviposition period (*r* = −0.901; *df* = 12; *p* < 0.001), fecundity (*r* = −0.894; *df* = 12; *p* < 0.001), female longevity (*r* = −0.739; *df* = 12; *p* = 0.003), male longevity (*r* = −0.739; *df* = 12; *p* = 0.003), net reproductive rate (*R*) (*r* = −0.815; *df* = 12; *p* < 0.001), and intrinsic rate of increase (*r*) (*r* = −0.947; *df* = 12; *p* < 0.001), but exhibited a strong positive correlation with mean generation time (*T*; 98%) (*r* = 0.982; *df* = 12; *p* < 0.001). Fecundity was negatively associated with development time (*r* = −0.894; *df* = 12; *p* < 0.001) and *T* (*r* = −0.889; *df* = 12; *p* < 0.001), while showing positive correlations with survival (*r* = 0.721; *df* = 12; *p* = 0.004), oviposition period (*r* = 0.782; *df* = 12; *p* < 0.001), female longevity (*r* = 0.733; *df* = 12; *p* = 0.003), male longevity (*r* = 0.697; *df* = 12; *p* = 0.006), *R*_0_ (*r* = 0.936; *df* = 12; *p* < 0.001), and *r* (*r* = 0.964; *df* = 12; *p* < 0.001). Similarly, the intrinsic rate of increase (*r*) was strongly and negatively correlated with development time (*r* = −0.947; *df* = 12; *p* < 0.001) and *T* (*r* = −0.946; *df* = 12; *p* < 0.001), and positively correlated with survival (*r* = 0.731; *df* = 12; *p* = 0.003), oviposition period (*r* = 0.826; *df* = 12; *p* < 0.001), fecundity (*r* = 0.964; *df* = 12; *p* < 0.001), female longevity (*r* = 0.736; *df* = 12; *p* = 0.003), male longevity (*r* = 0.716; *df* = 12; *p* = 0.004), and *R*_0_ (*r* = 0.919; *df* = 12; *p* < 0.001). In contrast, no significant correlations were detected between the finite rate of increase (*λ*) and the other demographic parameters.

**Figure 4:**
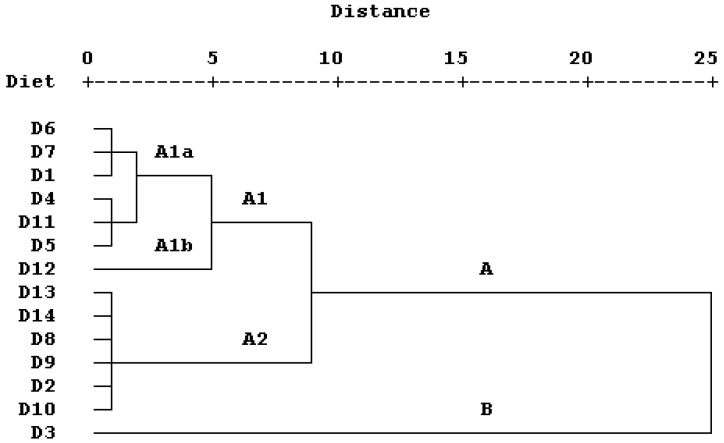
Dendrogram illustrating the relatedness among experimental diets based on demographic parameters of *Leucania loreyi* reared on maize- and cowpea seed-based artificial diets supplemented with different pollen sources, constructed using Ward’s minimum-variance method. Diets D1–D6 consisted of maize seed combined with rapeseed, date palm, maize, common hollyhock, saffron, and honey bee pollens, respectively. Diets D8–D13 consisted of cowpea seed combined with the same sequence of pollen sources. Diets D7 and D14 contained maize and cowpea seeds alone, respectively.

Hierarchical cluster analysis of the demographic parameters of *L. loreyi* reared on maize- and cowpea seed-based artificial diets supplemented with different pollen sources revealed two primary clusters, designated A and B (Figure 4). Cluster A was further divided into two subclusters, A1 and A2. Subcluster A1 separated into two distinct groups: group *a*, which included diets D6 (maize seed–honey bee pollen diet), D7, D1, D4 (maize seed–common hollyhock pollen diet), D11, and D5; and group *b*, which consisted solely of diet D12. Subcluster A2 comprised diets D13, D14, D8 (cowpea seed–rapeseed pollen diet), D9 (cowpea seed–date palm pollen diet), D2, and D10.

## 4. Discussion

In the present study, incorporating maize and cowpea seeds together with diverse pollen sources into artificial diets significantly influenced the demographic performance of *L. loreyi* larvae. Diets formulated solely with maize seeds (D7) or cowpea seeds (D14) were sufficient to meet the basic nutritional requirements of *L. loreyi*, supporting complete development and survival. A similar outcome was reported by Ge et al. [33], who successfully reared *S. frugiperda* larvae on an artificial diet formulated exclusively with wheat bran. Nevertheless, artificial diets combining both seed and pollen ingredients may further improve nutritional balance by providing complementary nutrient profiles or by diluting plant-derived secondary compounds, thereby maximizing insect performance [34,35,36]. Mixed diets have also been used successfully in laboratory rearing of other insect species [33,37]. For example, Kefayat et al. [16,19] supplemented cowpea seed-based artificial diets with various pollen sources—including honey bee, glossy shower, maize, hollyhock, date palm, saffron, sunflower, and rapeseed—and found that diets containing date palm pollen supported the highest performance in *H. armigera*. Similarly, Hemmati et al. [24] developed seed-based artificial diets incorporating date palm pollen and reported that cowpea- and mung bean seed–date palm pollen diets were optimal for rearing *Spodoptera littoralis* (Boisduval) (Lepidoptera: Noctuidae). Collectively, these findings demonstrate that seed and pollen sources are not nutritionally equivalent and can differentially affect insect development, survival, and reproduction.

The results of the present study demonstrated that diets D3 (maize seed–maize pollen diet) and D10 (cowpea seed–maize pollen diet) supported more rapid development of *L. loreyi*, as evidenced by shorter larval and pupal durations on both diets. These findings indicate that the enhanced performance observed on these diets was primarily attributable to the inclusion of maize pollen rather than to differences in the seed components used as the dietary base. Maize pollen is known to contain high levels of essential nutrients, including crude proteins, carbohydrates, lipids, fibers, and minerals such as sodium, magnesium, calcium, zinc, and iron [38], while exhibiting relatively low concentrations of secondary metabolites, such as phenolic compounds [16].

The developmental duration of *L. loreyi* on diets D3 and D10 was comparable to that reported by Kefayat et al. [19] for *H. armigera* reared on a cowpea seed-based diet supplemented with maize pollen (37.2 days), and by Hemmati et al. [24] for *S. littoralis* reared on a maize seed-based diet enriched with date palm pollen (38.6 days). However, development of *L. loreyi* on diet D10 was faster than that reported for *S. littoralis* reared on a cowpea seed-based diet supplemented with date palm pollen (44.3 days) [24]. Despite similarities in the basal diet formulation, such discrepancies may reflect differences among insect species, the specific pollen types incorporated, and the nutritional profiles of those pollens.

In contrast, diets D5 (maize seed–saffron pollen diet) and D12 (cowpea seed–saffron pollen diet) resulted in prolonged developmental periods, indicating their lower suitability for *L. loreyi* rearing. This effect is likely attributable to the inclusion of saffron pollen rather than to differences between seed components. Pollen grains vary widely in size, structural characteristics (e.g., shape and surface morphology), digestibility, and nutritional composition [38]. It is plausible that the thick exine layer of saffron pollen [39], elevated concentrations of secondary metabolites [40], and/or relatively low levels of primary metabolites [16], limited nutrient availability in diets D5 and D12. Notably, high levels of secondary metabolites and reduced nutritional quality have previously been identified as key factors constraining *L. loreyi* performance on maize hybrids [4].

The results of this study indicate that the tested artificial diets substantially influenced the reproductive performance of *L. loreyi*. In particular, females reared on diet D3 exhibited shorter adult pre-oviposition (APOP) and total pre-oviposition (TPOP) periods compared with those reared on the other diets, suggesting that reproductive maturation was accelerated on this formulation. Moreover, fecundity on diet D3 was 2.4-fold higher than that of individuals reared on diet D12, underscoring the superior suitability of diet D3 for enhancing reproductive output. Successful reproduction in insects is generally associated with access to protein-rich food resources [41], and increased availability of dietary protein has been shown to enhance reproductive traits and promote population growth in insect pests [19,42]. Accordingly, the combination of maize seed and maize pollen in diet D3 appears to have provided a nutritionally balanced diet that fully satisfied the reproductive requirements of *L. loreyi*, resulting in higher fecundity than observed on unsupplemented seed-based diets (D7 and D14) or diets incorporating other seed or pollen sources. Consistent with the correlation analysis, fecundity was negatively associated with developmental duration and positively correlated with oviposition period and female longevity. Thus, the shorter developmental time, together with the prolonged oviposition period and increased adult female longevity observed on diet D3, likely contributed directly to the elevated fecundity recorded for females reared on this diet. Similar trends have been reported previously; He et al. [1] showed that fecundity of *L. loreyi* females increased when adults were provided with nutritionally suitable pollen diets, including sunflower, motherwort, rapeseed, lotus, pine, schisandra, and maize pollen solutions. Comparable effects have also been documented in other noctuid species. Hemmati et al. [24] reported that rearing *S. littoralis* larvae on cowpea- and mung bean seed-based artificial diets supplemented with date palm pollen prolonged oviposition period and adult longevity, leading to enhanced reproductive performance. In *H. armigera*, fecundity reached ~1140 offspring per female when larvae were reared on a cowpea seed-based diet supplemented with date palm pollen [19], a value substantially higher than the maximum fecundity observed in the present study (~800 offspring per female on diet D3). These differences are likely driven by interspecific variation in nutritional requirements and digestive physiology, including differences in digestive enzyme activity and tolerance to plant protease inhibitors and secondary metabolites, in addition to variation in the nutritional quality of the seed and pollen sources used across studies.

Based on the present results, the intrinsic rate of increase (*r*) of *L. loreyi* was highest on diet D3, indicating superior overall demographic performance on this formulation. Among life table parameters, *r* integrates the combined effects of diet on insect fitness [19,43], a pattern supported here by its strong negative correlations with developmental duration and mean generation time (*T*), and positive associations with survival, fecundity, oviposition period, adult longevity, and net reproductive rate (*R*_0_). Similar relationships between *r* and other life table parameters have been reported previously [44]. The range of *r* values obtained for *L. loreyi* in this study is comparable to those reported for the same species reared on artificial diets (0.118–0.125 day^−1^; [10]) and maize hybrids (0.083–0.133 day^−1^; [4]), as well as for *S. littoralis* reared on seed-based diets supplemented with date palm pollen (0.059–0.132 day^−1^; [24]). However, these values were lower than those reported for *L. loreyi* under varying temperature regimes (0.058–0.155 day^−1^; [45]) and for *H. armigera* reared on pollen-supplemented artificial diets (0.109–0.147 day^−1^; [19]), differences that likely reflect variation in insect species, diet composition, pollen type, and experimental conditions. In addition to exhibiting the highest *r*, insects reared on diet D3 showed higher *R*_0_ and finite rate of increase (*λ*), along with a shorter *T*, further confirming the high nutritional value of this diet. As maize is a preferred host plant for *L. loreyi* [3], its seeds and pollen likely provide a favorable nutrient profile that supports population growth, particularly given their high protein and starch contents and low phenolic levels [16,24]. It is worth noting that the *λ* represents population growth across successive age intervals and integrates survival and fecundity across the entire life span, whereas *r* is more sensitive to shifts in the timing of reproduction, particularly early in adulthood. Consequetly, *λ* may be less responsive to variation in individual demographic traits, which could explain the lack of significant correlations observed in this study. In contrast, diets D5 and D12 resulted in the lowest *R*_0_ (D12) and reduced *r* and *λ* values, accompanied by prolonged generation times, suggesting constrained population growth on these formulations. The inclusion of saffron pollen—characterized by relatively low levels of protein, lipids, and starch [16]—appears to be a major factor underlying these effects. Although dietary protein is generally associated with enhanced insect performance and population growth [16,46,47,48], *L. loreyi* exhibited slower population increase on saffron pollen–supplemented diets than on unsupplemented seed-based diets (D7 and D14) or diets containing other pollen sources. This finding suggests that multiple protein sources, particularly at elevated levels, may not be efficiently utilized by larvae, as previously reported for *Anastrepha ludens* (Loew) (Diptera, Tephritidae) [49], and that nutrient imbalances or antagonistic interactions among plant-derived components may limit diet suitability, underscoring the importance of evaluating overall nutritional composition rather than individual ingredients.

Cluster analysis grouped the experimental artificial diets according to their overall suitability for *L. loreyi*. Diet D3, which formed a distinct cluster (cluster B), consistently supported superior performance across developmental, reproductive, and population growth parameters, whereas diet D12 (subcluster A1b) exhibited an opposing pattern with reduced demographic performance. The superior demographic performance observed on diet D3 may reflect the nutritional suitability of maize pollen for lepidopteran larvae, particularly its contribution of essential amino acids needed for ovarian development, sterols required for ecdysteroid synthesis, and vitamins that support growth, reproduction, and metabolism [1,50,51]. In contrast, the poorer performance on diet D12, which contained saffron pollen, may indicate nutritional imbalance and/or the presence of secondary metabolites that reduce digestion efficiency or nutrient assimilation [15,16,40]. Because we did not directly quantify pollen nutritional composition, these interpretations are necessarily inferential and based on general principles of insect nutrition rather than direct evidence from this study. Diets containing honey bee pollen (D6 and D13) showed intermediate demographic performance, but pollen-specific effects should be interpreted cautiously because the botanical composition of honey bee pollen is typically unknown and can vary. Similar clustering of artificial diets based on demographic performance has been reported for other noctuid species reared on plant-derived diet formulations [18,19,24].

## 5. Conclusions

The results of this study demonstrate that supplementation of maize- and cowpea seed-based artificial diets with pollen sources markedly influences the demographic performance of *L. loreyi*. Rearing insects on the lower-quality diet D12 prolonged the incubation and pupal periods, as well as the adult and total pre-oviposition periods, and reduced fecundity, female longevity, net reproductive rate (*R*_0_), intrinsic rate of increase (*r*), and gross reproductive rate (*GRR*), indicating that saffron pollen has limited nutritional suitability as a dietary supplement. In contrast, diet D3 was identified as a high-quality formulation, as it accelerated development and significantly enhanced key demographic parameters, including oviposition period, fecundity, female longevity, *R*_0_, *r*, *GRR*, and finite rate of increase (*λ*). Overall, these findings identify maize seed and maize pollen as nutritionally favorable plant-derived components for *L. loreyi* artificial diets under controlled conditions and highlight their potential for future diet optimization. However, additional studies are needed to evaluate economic feasibility, diet stability, and scalability before adoption in large-scale rearing systems or integrated pest management (IPM) programs.

## Figures and Tables

**Table 1:** Composition of maize- and cowpea seed-based artificial diets supplemented with diverse pollen sources for rearing *Leucania loreyi* larvae.

Diet	Base Ingredient (g)	Pollen Source (g)	Additional Components (g or mL)
D1	Maize (19.5)	Rapeseed (1)	wheat germ (3), yeast (3.5), ascorbic acid (0.35), sorbic acid (0.11), formaldehyde (0.25), methyl-p-hydroxy benzoate (0.22), agar (1.4), sunflower oil (0.5 mL), distilled water (65 mL)
D2	Maize (19.5)	Date palm (1)	Same as D1 *
D3	Maize (19.5)	Maize (1)	Same as D1 *
D4	Maize (19.5)	Common hollyhock (1)	Same as D1 *
D5	Maize (19.5)	Saffron (1)	Same as D1 *
D6	Maize (19.5)	Honey bee (1)	Same as D1 *
D7	Maize (20.5)	No pollen	Same as D1 *
D8	Cowpea (19.5)	Rapeseed (1)	Same as D1 *
D9	Cowpea (19.5)	Date palm (1)	Same as D1 *
D10	Cowpea (19.5)	Maize (1)	Same as D1 *
D11	Cowpea (19.5)	Common hollyhock (1)	Same as D1 *
D12	Cowpea (19.5)	Saffron (1)	Same as D1 *
D13	Cowpea (19.5)	Honey bee (1)	Same as D1 *
D14	Cowpea (20.5)	No pollen	Same as D1 *

* All diets contained identical amounts of wheat germ, yeast, preservatives, agar, and distilled water.

**Table 2:** Development and survival (mean ± SE) of *Leucania loreyi* reared on maize- and cowpea seed-based artificial diets supplemented with pollens from various sources.

Diet	Incubation Period (Days)	Larval Period (Days)	Pre-Pupal Period (Days)	Pupal Period (Days)	Development Time (Days)	Pre-Adult Survival
D1	5.10 ± 0.006 abc	22.21 ± 0.16 b	2.47 ± 0.117 a	14.05 ± 0.19 ab	43.68 ± 0.29 bc	0.38 ± 0.07 bc
D2	4.89 ± 0.005 def	20.50 ± 0.11 d	2.09 ± 0.006 a	12.75 ± 0.14 d	40.05 ± 0.22 f	0.40 ±0.07 bc
D3	4.81 ± 0.006 f	18.76 ± 0.12 e	1.10 ± 0.005 a	12.10 ± 0.12 e	37.53 ± 0.18 g	0.60 ± 0.07 a
D4	4.84 ± 0.009 ef	22.46 ± 0.41 b	2.72 ± 0.108 a	13.53 ± 0.21 bc	43.30 ± 0.71 bcd	0.34 ± 0.07 bc
D5	5.12 ± 0.006 ab	24.44 ± 0.22 a	2.39 ± 0.117 a	14.11 ± 0.22 ab	45.82 ± 0.34 a	0.34 ± 0.07 bc
D6	5.12 ± 0.008 abc	22.07 ± 0.14 b	2.09 ± 0.006 a	14.00 ± 0.16 ab	42.78 ± 0.21 cd	0.46 ± 0.07 abc
D7	4.92 ± 0.007 cef	21.63 ± 0.19 bc	2.98 ± 0.61 a	14.05 ± 0.18 ab	43.35 ± 0.69 bcd	0.38 ± 0.07 bc
D8	5.00 ± 0.007 abcde	21.75 ± 0.15 bc	2.15 ± 0.007 a	13.68 ± 0.19 bc	42.24 ± 0.33 d	0.50 ± 0.07 ab
D9	5.14 ± 0.008 ab	21.75 ± 0.22 bc	2.16 ± 0.007 a	13.84 ± 0.15 b	42.60 ± 0.26 d	0.50 ± 0.07 ab
D10	4.98 ± 0.053 bcde	21.81 ± 0.13 e	2.04 ± 0.004 a	12.18 ± 0.19 e	38.10 ± 0.30 g	0.44 ± 0.07 abc
D11	4.86 ± 0.005 ef	19.11 ± 0.21 b	2.47 ± 0.131 a	14.07 ± 0.18 ab	43.53 ± 0.39 bc	0.30 ± 0.06 c
D12	5.22 ± 0.009 a	22.16 ± 0.17 a	2.32 ± 0.109 a	14.33 ± 0.11 a	45.56 ± 0.30 a	0.36 ± 0.07 bc
D13	4.98 ± 0.004 bcde	23.95 ± 0.40 cd	2.06 ± 0.006 a	13.23 ± 0.16 c	41.06 ± 0.37 e	0.34 ± 0.07 bc
D14	5.02 ± 0.005 abcd	20.82 ± 0.18 d	2.00 ± 0.008 a	13.33 ± 0.21 bc	40.94 ± 0.33 e	0.36 ± 0.07 bc

Means followed by different letters in each column are significantly different (*p* < 0.05, Paired bootstrap test). Diets D1–D6 consisted of maize seed combined with rapeseed, date palm, maize, common hollyhock, saffron, and honey bee pollens, respectively. Diet D8–D13 consisted of cowpea seed combined with the same sequence of pollen sources. Diets D7 and D14 contained maize and cowpea seeds alone, respectively.

**Table 3:** Reproduction, longevity, and total lifespan (mean ± SE) of *Leucania loreyi* reared on maize- and cowpea seed-based artificial diets supplemented with pollen from various sources.

Diet	APOP (Days)	TPOP (Days)	Oviposition Period (Days)	Fecundity (Offspring)	Longevity (Days)		Whole Lifespan (Days)	
					Female	Male	Female	Male
D1	4.91 ± 0.16 a	47.91 ± 0.47 c	9.28 ± 0.56 de	509.39 ± 43.57 cdef	16.81 ± 0.23 de	15.24 ± 0.60 bc	59.82 ± 0.41 bc	59.86 ± 0.94 a
D2	4.41 ± 0.14 ab	44.00 ± 0.30 f	10.92 ± 0.50 abc	550.71 ± 25.41 bcd	18.00 ± 0.63 bcd	14.89 ± 0.80 bc	57.58 ± 0.64 def	55.63 ± 0.94 ab
D3	3.78 ± 0.10 d	41.05 ± 0.21 g	11.89 ± 0.28 a	801.69 ± 42.42 a	19.33 ± 0.43 a	17.18 ± 0.40 ab	56.61 ± 0.41 ef	55.09 ± 0.60 b
D4	4.72 ± 0.31 ab	48.17 ± 1.03 bc	9.91 ± 0.37 cde	471.45 ± 31.63 def	18.37 ± 0.62 abc	14.81 ± 1.22 bcd	61.82 ± 0.69 a	57.76 ± 2.90 ab
D5	4.91 ± 0.25 ab	50.45 ± 0.43 a	9.09 ± 0.31 e	432.77 ± 16.97 f	15.91 ± 0.54 ef	11.16 ± 0.50 e	61.45 ± 0.74 ab	57.44 ± 2.04 ab
D6	4.18 ± 0.18 bc	46.37 ± 0.39 cd	10.17 ± 0.48 bcde	515.88 ± 24.23 cde	18.27 ± 0.54 abc	16.58 ± 0.64 abc	60.45 ± 0.69 abc	59.91 ± 0.65 a
D7	4.46 ± 0.16 ab	47.98 ± 1.11 bc	10.35 ± 0.51 bcd	517.81 ± 37.70 cde	17.18 ± 0.29 cde	14.76 ± 1.00 cd	60.70 ± 1.32 abc	57.88 ± 1.35 ab
D8	4.30 ± 0.15 bc	46.00 ± 0.74 cde	10.50 ± 0.58 bcd	554.34 ± 25.78 bc	18.20 ± 0.41 abc	15.73 ± 0.49 bc	59.90 ± 0.50 bc	58.33 ± 0.56 ab
D9	4.46 ± 0.21 ab	46.18 ± 0.37 cd	10.00 ± 0.50 cde	548.42 ± 42.87 bcde	18.09 ± 0.31 bc	15.93 ± 0.70 abc	59.82 ± 0.41 bc	59.21 ± 0.72 ab
D10	3.85 ± 0.10 cd	41.16 ± 0.30 g	11.23 ± 0.34 ab	612.98 ± 25.59 b	18.54 ± 0.58 ab	17.77 ± 0.59 a	55.85 ± 0.69 f	57.00 ± 0.64 ab
D11	4.70 ± 0.33 ab	47.90 ± 0.64 c	10.00 ± 0.25 cde	468.66 ± 21.05 ef	18.50 ± 0.56 ab	15.91 ± 1.44 abc	61.70 ± 0.63 a	59.85 ± 4.56 ab
D12	4.90 ± 0.23 a	50.20 ± 0.44 ab	10.10 ± 0.40 cde	339.73 ± 32.50 g	15.60 ± 0.39 f	12.24 ± 0.56 de	60.90 ± 0.40 ab	58.11 ± 0.71 ab
D13	4.36 ± 0.24 abc	45.09 ± 0.67 def	10.55 ± 0.34 bcd	568.03 ± 22.91 bc	17.37 ± 0.59 bcde	13.83 ± 1.46 cde	58.09 ± 0.76 cde	55.43 ± 2.91 ab
D14	4.45 ± 0.20 ab	45.09 ± 0.35 ef	10.73 ± 0.46 bc	577.10 ± 27.58 bc	17.64 ± 0.43 bcd	16.27 ± 1.12 abc	58.27 ± 0.30 cd	57.69 ± 1.52 ab

Means followed by different letters within a column differ significantly (*p* < 0.05; paired bootstrap test). APOP = adult pre-oviposition period; TPOP = total pre-oviposition period. Diets D1–D6 consisted of maize seed combined with rapeseed, date palm, maize, common hollyhock, saffron, and honey bee pollens, respectively. Diets D8–D13 consisted of cowpea seed combined with the same sequence of pollen sources. Diets D7 and D14 contained maize and cowpea seeds alone, respectively.

**Table 4:** Life table parameters (mean ± SE) of *Leucania loreyi* reared on maize- and cowpea seed-based artificial diets supplemented with pollen from various sources.

Diet	*R*_0_ (Offspring)	*r* (Day^−1^)	*GRR* (Offspring)	*λ* (Day^−1^)	*T* (Day)
D1	111.89 ± 31.28 bc	0.089 ± 0.006 cd	525.23 ± 189.69 a	1.093 ± 0.006 cd	52.47 ± 0.57 b
D2	132.36 ± 33.70 bc	0.099 ± 0.006 bcd	341.38 ± 63.70 ab	1.105 ± 0.006 bcd	48.81 ± 0.26 e
D3	288.691±55.944 a	0.122 ± 0.004 a	485.23 ± 76.87 a	1.130 ± 0.005 a	46.14 ± 0.35 f
D4	103.51 ± 28.59 bc	0.087 ± 0.006 cd	322.53 ± 58.68 ab	1.092 ± 0.007 cd	52.30 ± 1.28 bc
D5	95.33 ± 25.70 bc	0.082 ± 0.005 d	310.92 ± 48.30 abc	1.086 ± 0.006 d	54.82 ± 0.43 a
D6	113.10 ± 30.42 bc	0.092 ± 0.006 cd	247.85 ± 55.44 bc	1.097 ± 0.006 cd	50.85 ± 0.35 bc
D7	114.05 ± 31.21 bc	0.089 ± 0.006 cd	553.39 ± 201.48 a	1.094 ± 0.006 cd	52.51 ± 0.90 b
D8	111.29 ± 31.65 bc	0.093 ± 0.006 cd	225.08 ± 56.06 bc	1.907 ± 0.007 cd	50.27 ± 0.82 bcde
D9	120.69 ± 33.55 bc	0.099 ± 0.005 bcd	242.00 ± 58.49 bc	1.103 ± 0.489 bcd	50.62 ± 0.34 bcd
D10	159.14 ± 38.37 ab	0.110 ± 0.006 b	362.72 ± 66.63 ab	1.116 ± 0.006 b	45.95 ± 0.37 f
D11	93.52 ± 26.74 bc	0.086 ± 0.006 cd	315.01 ± 60.41 abc	1.090 ± 0.007 cd	52.40 ± 0.56 b
D12	68.15 ± 19.88 c	0.076 ± 0.006 d	222.12 ± 45.08 c	1.079 ± 0.006 d	55.00 ± 0.37 a
D13	125.07 ± 33.94 bc	0.097 ± 0.006 bcd	368.33 ± 68.11 ab	1.102 ± 0.007 bcd	49.51 ± 0.50 de
D14	126.98 ± 34.36 bc	0.096 ± 0.006 bcd	359.01 ± 70.18 ab	1.101 ± 0.006 bcd	49.51 ± 0.50 de

Means followed by different letters within a column differ significantly (*p* < 0.05; paired bootstrap test). *R*_0_ = net reproductive rate; *r* = intrinsic rate of increase; *GRR* = gross reproductive rate; *λ* = finite rate of increase; *T* = mean generation time. Diets D1–D6 consisted of maize seed combined with rapeseed, date palm, maize, common hollyhock, saffron, and honey bee pollens, respectively. Diets D8–D13 consisted of cowpea seed combined with the same sequence of pollen sources. Diets D7 and D14 contained maize and cowpea seeds alone, respectively.

## Data Availability

Data are contained within the article.

## Abbreviations

The following abbreviations are used in this manuscript:

APOP	Adult pre-oviposition period
TPOP	Total pre-oviposition period
*R*_0_	Net reproductive rate
*r*	Intrinsic rate of increase
*GRR*	Gross reproductive rate
*λ*	Finite rate of increase
*T*	Mean generation time

## References

[1] He L., Zhao S., He W., Wu K. (2022). Pollen and nectar have different effects on the development and reproduction of noctuid moths. Front. Ecol. Evol..

[2] Ben Jemâa J.M., Soltani A., Djebbi T., Mejri I., Kanyesigye D., Otim M.H. (2023). The maize caterpillar *mythimna* (=*Leucania*) *loreyi* (Duponchel, 1827) (Lepidoptera: Noctuidae): Identification, distribution, population density and damage in Tunisia. Insects.

[3] El-Sherif S.I., Hammad S.M., El-Sawaf S.K. (2009). Field observations on *Leucania loreyi* (Dup.) (Lepid., Noctuidae) in Egypt. J. Appl. Entomol..

[4] Jafari H., Hemmati S.A., Esfandiari M., Rasekh A. (2025). Population growth, digestive physiology, and antioxidant enzyme activities of *Leucania loreyi* (Lepidoptera: Noctuidae) on maize hybrids. Crop Prot..

[5] Song H.Y., Li L.L., Zhang Q.Q., Song Y.Y., Zhu Z.G., Lu Z.B., Yu Y., Men X. (2021). Southward migration routes of insect species in Shandong Province. Chin. J. Appl. Entomol..

[6] Baek S., Kim M.J., Kim E.Y., Jung J.K., Park C.G. (2024). Assessment of the occurrence of the second generation of *Mythimna loreyi* duponchel (Lepidoptera: Noctuidae) using temperature-dependent developmental and oviposition models. PLoS ONE.

[7] He L., Sun X., Tan Y., Zhou Y., Wu W., Liu X., Wu K. (2026). Flight capacity and wingbeat frequency of *Mythimna loreyi* (Lepidoptera: Noctuidae). Environ. Entomol..

[8] Ebrahimi L., Amir-Maafi M., Shiri M. (2024). Investigating the effect of temperature on abundance of *Mythimna loreyi* (Lepidoptera: Noctuidae) in corn. J. Iran. Plant Prot. Res..

[9] Ravan B., Esfandiari M., Mossadegh M.S. (2024). First record of *Leucania herrichii* Herrich-Schäffer from Iran with new distribution data of Leucaniini (Lep., Noctuidae). J. Insect Biodivers. Syst..

[10] Kim E.Y., Kim I.H., Jung J.K. (2022). Developmental and reproductive characteristics of *Mythimna loreyi* (Noctuidae) reared on artificial diets. Korean J. Appl. Entomol..

[11] Jafari H., Habibpour B., Hemmati S.A., Stelinski L.L. (2023). Population growth parameters of *Helicoverpa armigera* (Hübner) on various legume seeds reveal potential tolerance traits. Sustainability.

[12] Zergani A., Shishehbor P., Naser Nakkai F., Riahi E. (2023). Life history traits and population parameters of the predatory mite *Euseius scutalis* (Acari: Phytoseiidae) fed on *Tetranychus turkestani* (Acari: Tetranychidae) and pollen from three different plants. Acarologia.

[13] Lukšic K., Mucalo A., Marinov L., Ozretic Zokovic M., Rankovic-Vasic Z., Nikolic D., Zdunic G. (2024). X-ray microanalysis of elemental composition of *Vitis sylvestris* pollen grains. Plants.

[14] Sobhy I.S., Gurr G.M., Jones T.H. (2024). Induced plant resistance and its influence on natural enemy use of plant-derived foods. Curr. Opin. Insect Sci..

[15] Arab Yabarati Z., Hemmati S.A., Esfandiari M., Siahpoosh M.R. (2025). Relationship between the nutritional and physiological responses of *Helicoverpa armigera* (Hübner) and phytochemical metabolites in various sesame cultivars. J. Appl. Entomol..

[16] Kefayat F., Hemmati S.A., Rasekh A., Nasernakhaei F., Stelinski L.L. (2025). Suitability of artificial diets containing various types of pollen grains to *Helicoverpa armigera* (Hübner, 1808): Nutritional performance and digestive enzyme response. Insects.

[17] Bakaze E., Kiggundu A. (2018). Use of artificial diets with plant material to evaluate banana cultivars for resistance to *Cosmopolites sordidus*. Uganda J. Agric. Sci..

[18] Arab Yabarati Z., Hemmati S.A., Esfandiari M., Siahpoosh M.R. (2025). Population growth performance and antioxidant enzymes activities of *Helicoverpa armigera* (Lepidoptera: Noctuidae) on diets from various sesame cultivars. J. Insect Sci..

[19] Kefayat F., Hemmati S.A., Rasekh A., Nasernakhaei F. (2025). The suitability of artificial diets containing diverse pollen grains to *Helicoverpa armigera* (Hübner): Population growth performance and antioxidant enzymes activities. J. Asia Pac. Entomol..

[20] Hayashida R., Bueno A.D.F., Hermel A.O., Hirakuri M.H., Silva F.A.C., Roggia S. (2018). *Euschistus heros* (Hemiptera: Pentatomidae) fitness on artificial diets: An approach to optimize mass rearing of *Telenomus podisi* (Hymenoptera: Platygastridae) for augmentative biological control. J. Econ. Entomol..

[21] Sayed W.A.A., El-Helaly A., Jamal Z.A., El-Bendary H. (2021). Effect of a low cost diet on the cotton leaf worm, *Spodoptera littoralis* nucleopolyhedrosis virus pathogenicity and sterile insect technique. Egypt. J. Biol. Pest Control.

[22] Tibola C.M., Silva L., Sgubin F., Omoto C. (2021). Monitoring resistance of *Euschistus heros* (Fabricius) (Hemiptera: Pentatomidae) to insecticides by using encapsulated artificial diet bioassay. Insects.

[23] Jung J.K., Kim E.Y., Kim I.H., Ahn J.J., Lee G.S., Seo B.Y. (2020). Meridic diets for rearing of *Spodoptera frugiperda* larvae. Korean J. Appl. Entomol..

[24] Hemmati S.A., Arab Yabarati Z., Stelinski L.L. (2025). Enrichment of diverse seed-based artificial diets with date palm pollen affects the fitness and physiological responses of *Spodoptera littoralis* (Boisduval, 1833). Sci. Rep..

[25] Shorey H.H., Hale R.L. (1965). Mass-rearing of the larvae of nine noctuid species on a simple artificial medium. J. Econ. Entomol..

[26] Jiang S., Sun X.T., Ge S.S., Yang X.M., Wu K.M. (2023). Mating competitiveness of male *Spodoptera frugiperda* (Smith) irradiated by X-rays. Insects.

[27] Huang Y.B., Chi H. (2013). Life tables of *Bactrocera cucurbitae* (Diptera: Tephritidae): With an invalidation of the jackknife technique. J. Appl. Entomol..

[28] Chi H. (1988). Life-table analysis incorporating both sexes and variable development rates among individuals. Environ. Entomol..

[29] Chi H., Su H.Y. (2006). Age-stage, two-sex life tables of *Aphidius gifuensis* (Ashmead) (Hymenoptera: Braconidae) and its host *Myzus persicae* (Sulzer) (Homoptera: Aphididae) with mathematical proof of the relationship between female fecundity and the net reproductive rate. Environ. Entomol..

[30] Chi H., Liu H. (1985). Two new methods for the study of insect population ecology. Bull. Inst. Zool. Acad. Sin..

[31] Chi H. (2022). TWOSEX-MSChart: A Computer Program for the Age-Stage, Two-Sex Life Table Analysis.

[32] Ward J.H. (1963). Hierarchical grouping to optimize an objective function. J. Am. Stat. Assoc..

[33] Ge S., Chu B., He W., Jiang S., Lv C., Gao L., Sun X., Yang X., Wu K. (2022). Wheat-bran-based artificial diet for mass culturing of the fall armyworm, *Spodoptera frugiperda* Smith (Lepidoptera: Noctuidae). Insects.

[34] Toft S., Wise D.H. (1999). Growth, development, and survival of a generalist predator fed single- and mixed-species diets of different quality. Oecologia.

[35] Singer M., Bernays E., Carriere Y. (2002). The interplay between nutrient balancing and toxin dilution in foraging by a generalist insect herbivore. Anim. Behav..

[36] Marques R.V., Sarmento R.A., Lemos F., Pedro-Neto M., Sabelis M.W., Venzon M., Pallini A., Janssen A. (2015). Active prey mixing as an explanation for polyphagy in predatory arthropods: Synergistic dietary effects on egg production despite a behavioural cost. Funct. Ecol..

[37] Ashok K., Balasubramani V., Kennedy J.S., Geethalakshmi V., Jeyakumar P., Sathiah N. (2021). Evaluating artificial diets for the fall armyworm, *Spodoptera frugiperda* (J.E. Smith) (Lepidoptera: Noctuidae) through nutritional indices and an age stage, two-sex life table approach. Afr. Entomol..

[38] Bujang J.S., Zakaria M.H., Ramaiya S.D. (2021). Chemical constituents and phytochemical properties of floral maize pollen. PLoS ONE.

[39] Sivaguru M., Mander L., Fried G., Punyasena S.W. (2012). Capturing the surface texture and shape of pollen: A comparison of microscopy techniques. PLoS ONE.

[40] Eggs B., Sanders D. (2013). Herbivory in spiders: The importance of pollen for orb-weavers. PLoS ONE.

[41] Wu K.J., Li M.H. (1993). Nutritional ecology of the cotton bollworm, *Heliothis armigera* (Hübner): Life tables of the population on the artificial diets with different protein levels. Acta Entomol. Sin..

[42] Samaras K., Pappas M.L., Fytas E., Broufas G.D. (2019). Pollen provisioning enhances the performance of *Amblydromalus limonicus* on an unsuitable prey. Front. Ecol. Evol..

[43] Jha R.K., Chi H., Tang L.C. (2012). A comparison of artificial diet and hybrid sweet corn for the rearing of *Helicoverpa armigera* (Hubner) (Lepidoptera: Noctuidae) based on life table characteristics. Environ. Entomol..

[44] Chamani M., Naseri B., Rafiee-Dastjerdi H., Emaratpardaz J., Farshbaf Pourabad R., Chenari Bouket A., Oszako T., Belbahri L. (2024). Examining innovative technologies: Nano-chelated fertilizers for management of wheat aphid (*Schizaphis graminum* Rondani). Insects.

[45] Qin J., Zhang L., Liu Y., Sappington T.W., Cheng Y., Luo L., Jiang X. (2017). Population projection and development of the *Mythimna loreyi* (Lepidoptera: Noctuidae) as affected by temperature: Application of an age-stage, two-sex life table. J. Econ. Entomol..

[46] Wang P., Furlong M.J., Walsh T.K., Zalucki M.P. (2019). Moving to keep fit: Feeding behavior and movement of *Helicoverpa armigera* (Lepidoptera: Noctuidae) on artificial diet with different protein: Carbohydrate ratios. J. Insect Sci..

[47] Truzi C.C., Vieira N.F., de Souza J.M., De Bortoli S.A. (2021). Artificial diets with different protein levels for rearing *Spodoptera frugiperda* (Lepidoptera: Noctuidae). J. Insect Sci..

[48] Lazarov S.B., Georgiev I.G., Atanasov A.Z., Hristakov I.S. (2026). Development of the hypopharyngeal glands of worker bees (*Apis mellifera* L.) when fed different protein sources during the spring period. Insects.

[49] Pascacio-Villafán C., Birke A., Williams T., Aluja M. (2017). Modeling the cost-effectiveness of insect rearing on artificial diets: A test with a tephritid fly used in the sterile insect technique. PLoS ONE.

[50] Pan X., Connacher R.P., O’Connor M.B. (2021). Control of the insect metamorphic transition by ecdysteroid production and secretion. Curr. Opin. Insect Sci..

[51] Force E., Dacher M., Debernard S. (2025). How the diet influences lepidopteran reproduction: Morpho-functional, behavioral, and endocrine aspects. J. Insect Physiol..

